# Unskillful Surgery

**Published:** 1835

**Authors:** H. G. Jameson

**Affiliations:** Professor in Cin. Med. Col.


### ART. II.—UNSKILLFUL SURGERY.

*A Brief Notice of a little bad Surgery, by a good Surgeon, wherein a Ligature was unskillfully applied.* Communicated by H. G. JAMESON, M. D., Professor in Cin. Med. Col.

We are told by Dupuytren, that an officer having a wound involving the temporal artery, and compression having been found ineffectual, this able surgeon cut down on the artery, and applied a ligature *on a roll of diachylon.*

Is there any difficulty in imagining that much pain, inflammation suppuration, and all these consequences having considerable duration, will follow such an operation? Is it good surgery, or is it bad surgery, to torment a patient with a remedy alike cruel and unnecessary? If this were a mere casual affair, of some ordinary practitioner, or less noted writer, we might leave it to pass for what it is worth; but, coming as it does from the highest authority, I feel an imperative call to invite attention to the case. I am the more disposed to notice this error of practice, because it seems as though the profession were benighted in regard to the choice of ligatures. Is it not enough that Physick, A. Cooper, Crampton, Travers, and many others have tested, and reported in favor of the dissoluble ligature; that the French and Germans have succeeded by the method of tortion, in saving their patients from much suffering; and especially that those who have tested the ligatures of animal skin, have found it not only safe, but more safe than the indissoluble ligatures. Is the experience of nearly twenty years, in the extensive use of the skin ligature, worth anything? If so, the present writer has, by his experiments upon living animals, and extensive application in surgical practice, shown the fitness; nay, the most decided superiority of these ligatures; and are they still unworthy of notice or imitation? Shall it be said that it is a matter of no consequence, whether we heal wounds by the first intention, over ligatures, which do not irritate and provoke unnecessary pain and suppuration; or apply cords, which, being indissoluble, fret the parts as would a thorn, or splinter of wood; and after causing much suffering, must divide whatever has been surrounded before they can come away. I have seen the case of a lady, where a surgeon, who stood high in his profession, suffered a thread ligature to get under the flap of integument raised in the amputation of a scirrus mamma; and being covered in, it caused painful suppuration, which lasted with much distress for eight months, and then found its way through the sound skin by ulceration. This lady assured me, she had suffered more from this ligature, a hundred fold, than she did from the operation for removal of the breast; and much deformity followed.

In the name of Heaven, I ask the profession, wherein can lie their objections to using ligatures, which afford them facilities and advantages, that cannot be had from any others, and which at the same time, relieve patients from a large portion of suffering, to say nothing of the greater safety of the skin ligatures over all others? I have now applied many hundreds of buckskin ligatures, to arteries of all sizes, from the common iliac, down to the smallest that require ligatures; to veins in cases of wounds and operations, when it was evident that hemorrhage which existed would be attended with danger, owing to the prostration of the patient, from great losses of blood. In every instance, the ligatures answered the end expected, and without ever, to the best of my knowledge and belief, increasing the suffering, or interrupting the healing of the wound. Indeed, it is an undeniable fact, that wounds can be healed without retardation with the skin ligature; with the indissoluble, of whatever material, they cannot be thus healed.

In arteries of the first order, I have clearly demonstrated, that the sides of the vessels cannot be brought into contact, without cutting through the coats at several points, owing to the coats being so dense, that they fall into plaits by which they are subject to be cut unequally — whereas by means of a soft yielding ligature, carefully and slowly drawn, we can render the approximation more complete, and without cutting the coats at all. I truly believe, that many of the failures where patients died from secondary hemorrhage, where one of the larger arteries was tied, were owing to the circumstance just named; and that had an animal ligature been applied, in a skillful manner, secondary hemorrhage would not have taken place. Is twenty years' extensive experience worth anything? If it is, I repeat the solemn and important truth, that I have never had secondary hemorrhage to occur in my practice, in all that time. Surely this cannot well be ascribed to mere chance.

May I ask again, why this apathy in a matter of vital importance? Nothing is more easily procured than a piece of buckskin in this country, or chamois or other soft dressed skin in all countries, free from any impregnation with tannin. I have uniformly used soft, thin pieces of deer skin, dressed as for gloves; and cutting this into narrow strips, I have a ligature which applies with great convenience; has the advantage of never slipping, so as to loosen the first knot while a second is tying; nor will it slip off the artery, if it has the least fair hold. It becomes imbued with the lymph of the blood before we are done tying it; and is thus closely agglutinated to the vessel to which it is tied. It in a few days becomes a pulpy mass, and is carried away by absorption. So perfectly compatible is this substance with the living structures, that no irritation whatever seems to arise from its presence; such indeed is its harmlessness, that when well applied, the coats of arteries to which it is applied are never cut through, on the contrary, the vasa vasorum are suffered to continue their work of circulation. Can there be any reasonable doubt, that there is less risk of secondary hemorrhage under such circumstances, than where a harsh, indissoluble ligature is applied, which is well known to fret the surface with which it comes in contact. But apart from the reasonableness of the matter-of-fact, there has been ample opportunity for ascertaining, by observation and experience, that the animal ligature is of incalculable advantage to surgery. To be most useful, these ligatures must not be rolled or made hard by rolling them, to make them round, as was recommended by Dr. Dorsey, in his System of Surgery; nor should they be stretched except very slightly, otherwise they will cut the coats of the artery, provided they are drawn pretty tight. The most ample experience leads me to conclude with Scarpa, that so far from there being any advantage in cutting the arterial coats, as taught by Dr. Jones, it is much better to direct our measures so as to avoid cutting the coats, or strangulating the vasa vasorum of the artery. In small arteries this is not a matter of much moment, but in those of the larger classes, it is of vast importance, since it will assuredly lessen the risk of secondary hemorrhage, to apply our ligatures so that they shall not cut the coats at all.

If it be a matter of so much interest to select the best ligature, what excuse may be offered for such a surgeon as Dupuytren, who stuffed in a roll of diachylon along with his indissoluble ligature? That such was the nature of the ligature there can be no doubt, else why use the plaster. Some of Dupuytren's cotemporaries, and many German surgeons, would have treated this artery by tortion. But why not apply the dissoluble ligature, which, while it secures the vessel, is perfectly harmless? I have again and again stayed the hemorrhage from wounded branches of the temporal and occipital arteries by tortion; those of the mamma, of the fingers and toes, hands, and feet, may be thus treated, provided we can get them sufficiently within our reach, where we cannot, the skin ligature may be applied with unerring certainty. Nothing but vanity can govern men, in blindly refusing to adopt useful expedients, because they are not the inventors of them.

